# Repetitive transcranial magnetic stimulation in central post-stroke pain: a meta-analysis and systematic review of randomized controlled trials

**DOI:** 10.3389/fnins.2024.1367649

**Published:** 2024-06-12

**Authors:** Ying Liu, Runqing Miao, Hui Zou, Qian Hu, Shao Yin, Fengya Zhu

**Affiliations:** ^1^Department of Acupuncture and Rehabilitation, Traditional Chinese Medicine Hospital of Renshou County, Meishan, China; ^2^Department of Preventive Treatment, Hospital of Chengdu University of Traditional Chinese Medicine, Chengdu, China; ^3^Department of Hematology, Meishan City People's Hospital, Meishan, China; ^4^School of Clinical Medicine, Hospital of Chengdu University of Traditional Chinese Medicine, Chengdu, China; ^5^Traditional Chinese Medicine Department, Zigong First People's Hospital, Zigong, China

**Keywords:** central pain, central post-stroke pain, repetitive transcranial magnetic stimulation, meta, meta-analysis, systematic reviews

## Abstract

**Background:**

The rehabilitation of central post-stroke pain (CPSP) is a complex clinical challenge, and repetitive transcranial magnetic stimulation (rTMS) has been widely applied in the research of neurofunctional recovery following stroke. However, there is currently no reliable evidence-based medicine supporting the efficacy of rTMS in central post-stroke pain. This review aims to evaluate the effects of rTMS on central post-stroke pain.

**Methods:**

Following the PRISMA guidelines, we conducted searches on PubMed, Cochrane Library, Embase, Web of Science, CNKI, and Wan Fang Data Knowledge Service Platform. We searched for randomized controlled trials (RCTs) investigating the use of rTMS in treating central post-stroke pain, and conducted screening based on inclusion and exclusion criteria. Characteristics of the included RCTs were extracted. The heterogeneity of the trials was assessed using the I2 statistic. Meta-analysis was performed using Stata 17 software. Bias risk and methodological quality were evaluated using the Cochrane RoB 2 tool and the Pedro scale.

**Results:**

A total of six randomized controlled trials involving 288 patients met our inclusion criteria. In our analysis, rTMS was more effective in treating patients with CPSP compared to the placebo group (SMD=-1.15, 95% CI: −1.69, −0.61, *P* < 0.001). Furthermore, results from subgroup analysis indicated no statistically significant difference in the improvement of pain for durations exceeding 6 months when comparing rTMS to conventional treatment (SMD=-0.80, 95% CI: −1.63, 0.03, *P* = 0.059).

**Conclusion:**

TMS can alleviate pain in CPSP patients and improve their motor function, but its effects on depression, anxiety, and MEP-latency are not significant.

**Systematic review registration:**

https://www.crd.york.ac.uk/prospero/, CRD42024497530.

## 1 Introduction

Stroke is one of the diseases with high global incidence, disability rates, and mortality rates (Zhang et al., 2020). Despite comprehensive rehabilitation treatments, most stroke patients experience varying degrees of recovery in motor and sensory functions. However, some patients still suffer from persistent pain on the affected side of the body after a stroke. This pain, occurring after a stroke and associated with the damaged area while excluding other causes, is referred to as CPSP (Radiansyah and Hadi, 2023). Although the onset time of CPSP may be related to the severity and progression of the condition, more than half of the cases manifest within the initial months following a stroke (Klit et al., 2009; Osama et al., 2018; Vukojevic et al., 2018). The incidence rate ranges from 1% to 35% (Dub and Mercier, 2011; Hansen et al., 2012). Many patients may experience various forms of pain concurrently with sensory abnormalities, such as searing, pressing, pulsating, or freezing sensations, numbness, and decreased sensation (Kumar, 2009; Klit et al., 2011). CPSP significantly impacts the sleep, emotions, and overall quality of life for stroke patients, hindering the implementation of effective rehabilitation treatments. The pathogenesis of CPSP is not fully understood, and its treatment remains challenging. Currently, the primary approach involves medications for neuropathic pain. Existing evidence suggests that even with the use of high-dose medications, pain relief is often difficult to achieve for the majority of CPSP patients (Scuteri et al., 2020; Singh et al., 2020; Choi et al., 2021; Mohanan et al., 2023). Additionally, these medications are associated with various side effects (Banerjee et al., 2013; Kim, 2014) and may lead to drug dependence (Kumar and Soni, 2009).

rTMS provides a non-invasive, painless method for studying and treating neuropathic pain states (Lefaucheur, 2016). By applying a magnetic field to the cerebral cortex, it induces electric currents, influencing neural electrical activity. This, in turn, regulates cerebral blood flow and neurotransmitter expression to alleviate pain. Currently, it is recommended by relevant treatment guidelines for various pain conditions (Winstein et al., 2016; Lefaucheur et al., 2020). In addition to its impact on the target area, the synaptic effects produced by rTMS contribute to its distal therapeutic effects (Hallett et al., 2017), but there is no uniform standard for therapeutic parameters in the treatment of CPSP using rTMS. Diverse treatment parameters, including stimulation frequency, target site, and duration of therapy, yield varying analgesic effects. Traditionally, low-frequency (LF) rTMS, defined as stimulation below 1 Hz, has been shown to reduce cortical excitability, whereas high-frequency (HF) rTMS, with frequencies above 1 Hz, exerts the opposite effect (Cruccu et al., 2007; Bai et al., 2022). Previous studies investigating the analgesic effects of rTMS on PSP have discovered that HF-rTMS (5–20 Hz) can effectively alleviate PSP-related pain (Pazzaglia et al., 2018). Compared to single and short-term interventions, multiple sessions and longer durations of intervention have been found to produce superior analgesic outcomes (Hosomi et al., 2013; Ramger et al., 2019).

The meta-analytic review conducted by McDonnell and Stinear (2017) indicated that, in stroke patients, the M1 of the non-affected hemisphere did not exhibit heightened activation during both active muscle contraction and rest, as evidenced by the absence of significant disparities in the parameters of aMT (active motor threshold), rMT (resting motor threshold), and MEPs (motor evoked potentials) when compared to those of healthy controls. This finding suggests that directly enhancing the excitability of the affected M1 may confer greater therapeutic benefits than indirectly suppressing the excitability of the unaffected M1 in facilitating motor recovery following stroke. Numerous previous studies have also discovered that LF-rTMS and continuous theta-burst stimulation (cTBS) not only suppress the amplitude of MEPs in the stimulated M1, but also enhance the MEP amplitude in the non-stimulated M1 (Di Lazzaro et al., 2011; Boddington and Reynolds, 2017). The increased cortical excitability within the unstimulated M1 may be associated with an elevated intrinsic excitability of excitatory interneurons responsible for glutamatergic non-NMDA receptor activity (Heide et al., 2006).

In studies utilizing a rat model of thalamic pain, it has been observed that neuronal structural damage occurs in the lesion area following cerebral hemorrhage or infarction, leading to increased neural excitability. Such alterations may precipitate a range of clinical manifestations, including limb pain and motor functional impairments (An et al., 2019). Other animal experiments have also demonstrated that CPSP reduces the functional connectivity between the VPL and S1/S2 (primary and secondary somatosensory cortices), responsible for perceiving pain location, intensity, and duration, while enhancing connectivity between the thalamus (involved in attention, cognitive abilities) and amygdala (associated with emotional aspects of pain assessment) (Sweet et al., 1971), rTMS can alleviate this abnormal connectivity (Gruart and Delgado-García, 1994).

In recent years, some reviews have summarized the impact of rTMS on pain (Pan et al., 2022; Cheng et al., 2023; Mohanan et al., 2023; Radiansyah and Hadi, 2023), suggesting that rTMS may have a beneficial effect in alleviating pain. However, some reviews primarily focus on exploring the mechanisms and concentrate on conditions such as fibromyalgia, postherpetic neuralgia, malignant neuropathic pain. There is limited analysis in these reviews regarding the clinical evidence of rTMS in treating CPSP. The effectiveness of rTMS for CPSP has not yet received sufficient support from evidence-based medicine. Therefore, to establish the relationship between rTMS and the relief of CPSP, we conducted a systematic review and meta-analysis of published randomized controlled trials. This meta-analysis aims to provide the latest evidence for the use of transcranial magnetic stimulation in the treatment of CPSP.

## 2 Methods

This study has been registered in PROSPERO with registration number CRD42023480458. Simultaneously, we will adhere to the PRISMA (Preferred Reporting Items for Systematic Reviews and Meta-analyses) guidelines to conduct and report the current systematic review and meta-analysis.

### 2.1 Eligibility criteria

#### 2.1.1 Study inclusion criteria

Participants: Individuals with a confirmed first-time stroke, whether ischemic or hemorrhagic, as verified by computed tomography (CT) or magnetic resonance imaging (MRI).

Confirmed CPSP Diagnosis: Participants must have a confirmed diagnosis of CPSP (Hansen et al., 2012; Scholz et al., 2019), exhibiting persistent or intermittent pain characterized by sensations of burning, throbbing, compression, or freezing (Klit et al., 2009).

Exclusion of Other Causes: Participants with CPSP excluding cases attributed to other diseases causing central neuropathic pain.

Intervention: Subjects undergoing rTMS as an intervention.

Comparison: The control group should receive either sham stimulation or conventional rehabilitation treatment. The specific interventions in the conventional rehabilitation treatment must be consistent with those in the intervention group.

Study Design: Randomized controlled trials with a crossover or parallel design.

#### 2.1.2 Exclusion criteria

Reviews, conference papers, animal studies, retrospective studies, case-control studies, and self-controlled studies will be excluded. Randomized controlled trials that do not report pain score-related outcomes will also be excluded.

### 2.2 Search strategy

We conducted searches in PubMed, Embase, Cochrane Library, Web of Science (WOS), Chinese National Knowledge Infrastructure (CNKI), and Wan Fang Data Knowledge Service Platform for relevant studies published until December 30, 2023. Additionally, manual searches of references in included studies and relevant reviews were performed to identify additional trials. Detailed search strategies and exclusion criteria can be found in Supplementary material.

### 2.3 Study selection

The search records obtained through the search strategy were imported into Endnote 21 to remove duplicate records. The first screening was conducted by reviewing titles and abstracts, followed by a full-text reading to determine the final inclusion of studies. Two reviewers (YL and QH) independently conducted the literature search and screening process. Any discrepancies between the two reviewers were resolved through discussion. If a consensus could not be reached, a third reviewer (FYZ) made the final decision.

### 2.4 Data extraction

Two reviewers independently conducted data extraction using a predefined standardized form. Extracted information included author and publication year, stroke onset time, sample size, participant demographics (age and gender), intervention details, relevant parameters, outcome indicators, and more. In cases where the original research data could not be obtained from the article, the corresponding author of the original study was contacted for the required information. After independent extraction, cross-checking was performed, and any discrepancies were resolved by the third reviewer (FYZ).

### 2.5 Assessment of risk of bias

The Cochrane RoB 2 tool was employed to assess the risk of bias in the included studies. The assessment covered five aspects of the study's overall risk of bias: randomization process, deviations from intended interventions, missing outcome data, measurement of the outcome, and selection of the reported result. For each randomized controlled trial (RCT), two reviewers (YL and QH) independently assessed each involved item as high risk, some concerns, or low risk. Discrepancies were resolved through verification. Additionally, the methodological quality was assessed using the Pedro scale. Any disagreements were consulted with a third reviewer (FYZ).

### 2.6 Outcome indicators

Primary outcome measures include Visual Analog Scale (VAS) or Numeric Rating Scale (NRS). Secondary outcome measures encompass McGill Pain Questionnaire (MPQ), Hamilton Rating Scale for Depression (HADM), Hamilton Anxiety Scale (HAMA), Motor Evoked Potential Latency (MEP-latency), and Fugl-Meyer Assessment for Upper Extremity (FMA-UE).

### 2.7 Data synthesis and statistical analysis

Statistical analysis was performed using Stata 17 software. Continuous data were expressed as standardized mean difference (SMD) with a 95% confidence interval (CI). In cases of substantial heterogeneity (I^2^ ≥ 50% or *P* < 0.05), a random-effects model was applied, and subgroup analyses were conducted to explore the sources of heterogeneity. Otherwise, a fixed-effects model was used. If I^2^ ≥ 75%, indicating “considerable heterogeneity,” sensitivity analysis was performed to assess result stability. A significance level of *P* < 0.05 was considered statistically significant for all analyses.

When multiple outcome measures were used in a study, the primary outcome measure reported in the article was prioritized for analysis.

## 3 Result

### 3.1 Results on literature search and selection

**Figure 1:**
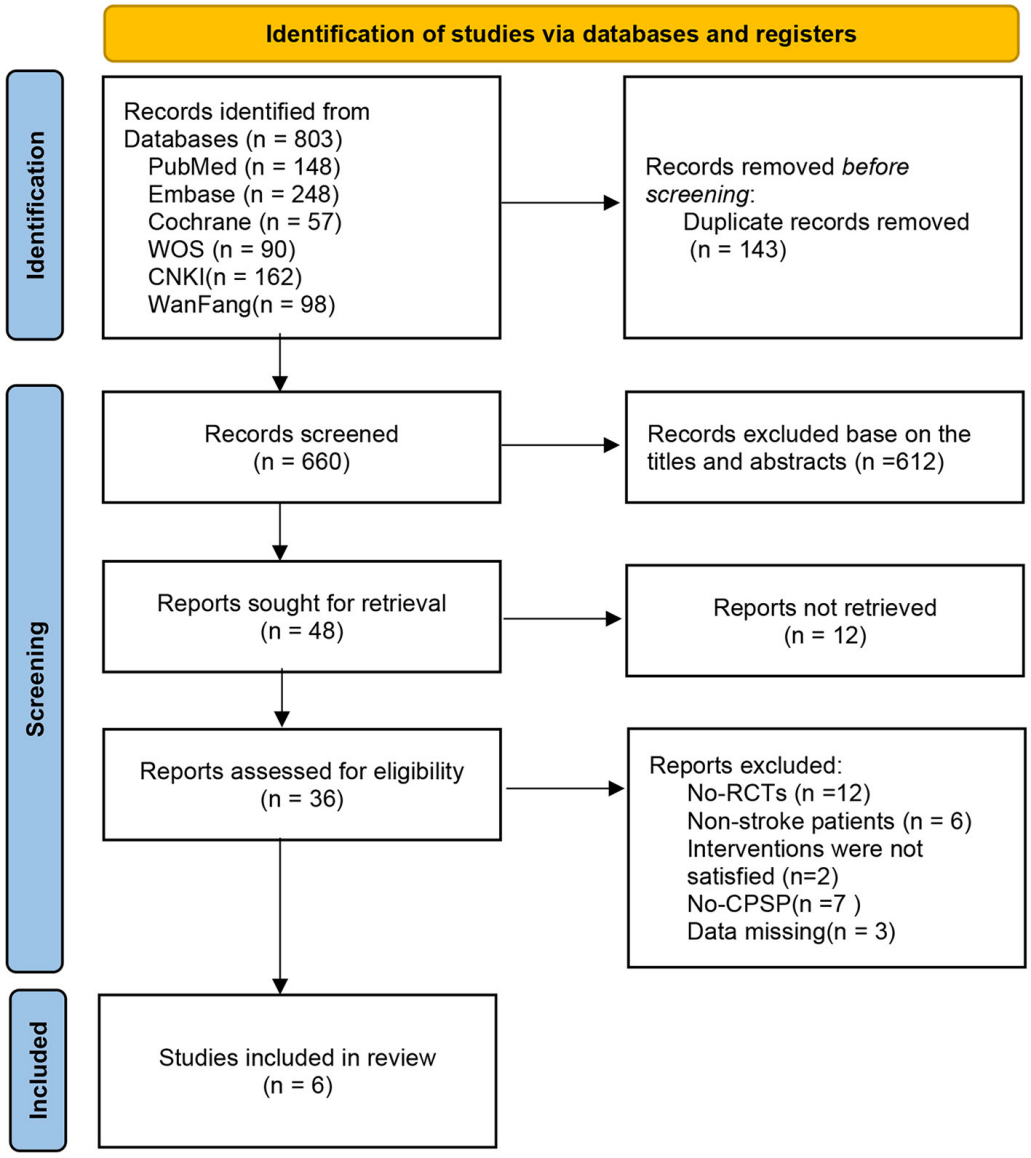
The screening flowchart.

We retrieved a total of 803 relevant articles from six databases, and after removing duplicates (143 articles), we evaluated them through title, abstract, and full-text reading. Finally, six eligible studies were included. A detailed flowchart is provided in Figure 1.

### 3.2 Characteristics of included study

This study included a total of six RCTs, comprising two English-language papers (De Oliveira et al., 2014; Zhao C.-G. et al., 2021) and four Chinese-language papers (Sun et al., 2019; Chen et al., 2020; Zhao Y. Y. et al., 2021; Jiang et al., 2022). The studies were published between 2014 and 2022, involving a total of 288 participants. Among them, 144 patients received rTMS combined with conventional rehabilitation training, while the remaining 144 patients underwent conventional rehabilitation training or sham stimulation. In the five studies (De Oliveira et al., 2014; Sun et al., 2019; Chen et al., 2020; Zhao C.-G. et al., 2021; Jiang et al., 2022) using sham stimulation combined with conventional rehabilitation training, four studies (De Oliveira et al., 2014; Sun et al., 2019; Zhao C.-G. et al., 2021; Jiang et al., 2022) employed a sham coil with no effective stimulation, and one study (Chen et al., 2020) used a method perpendicular to the surface of the skull for sham stimulation. Two studies (De Oliveira et al., 2014; Zhao Y. Y. et al., 2021) had a stroke duration >6 months, while the other four studies (Sun et al., 2019; Chen et al., 2020; Zhao C.-G. et al., 2021; Jiang et al., 2022) had a duration < 6 months. All studies assessed pain in patients, with 5 studies using VAS as the primary outcome measure. The study by Zhao C.-G. et al. (2021) used NRS. Three studies (Sun et al., 2019; Zhao C.-G. et al., 2021; Jiang et al., 2022) reported results for MEP-latency, and two studies (Chen et al., 2020; Zhao C.-G. et al., 2021) reported results for FMA-UE. The studies by Zhao C.-G. et al. (2021) and De Oliveira et al. (2014) reported results for MPQ, HAM-A, and HAM-D. For detailed characteristics, refer to Table 1.

**Table 1:** Characteristics of the randomized controlled studies.

**References**	**Study design**	**Year**	**Sample size (T, C)**	**Age [mean (SD)] (T, C)**	**Gender (male/ female) (T, C)**	**Duration of stroke (T/C)**	**Type of stroke (H:I) (T, C)**	**Intervention (T, C)**	**Coil type**	**Site**	**Treatment characteristics**	**Treatment time**	**Outcome indicator**
Jiang et al. (2022)	RCT	2022	32/32	61.56 ± 6.36, 60.13 ± 7.87	19/13, 17/15	33.25 ± 7.66 d, 32.81 ± 6.29 d	/	rTMS+CT, sham+CT	Figure-eight coil (MagPro R30 stimulator, Tonica Company, Denmark)	Ipsilesional M1	10 Hz, 80% RMT, 1500 pulses	8 weeks, 2 days per week	VAS, MEP
Zhao C.-G. et al. (2021)	RCT	2021	19,19	50.16 ± 11.34, 48.95 ± 11.51	/	12.21 ± 5.61 m,10.63 ±5.77 m	/	rTMS+CT, sham+CT	Figure-eight coil (CCY-1 stimulator, Yiruide Medical Equipment Company, China)	Ipsilesional M1	10 Hz, 80% RMT, 1500 pulses	3 weeks, 6 days per week	NRS, SF-MPQ-2, MEP, HAM-A HAM-D
Zhao Y. Y. et al. (2021)	RCT	2021	41/42	52.03 ± 14.22, 52.11 ± 14.28	25/16, 27/15	2.13 ± 0.51 m, 2.16 ± 0.52 m	20/21, 22/20	rTMS+CT, CT	Figure-eight coil (CCY-1 stimulator, Yiruide Medical Equipment Company, China)	Ipsilesional M1	10 Hz, 90% RMT, 1000 pulses	4 weeks, 7 days per week	VAS, FMA-UE
Chen et al. (2020)	RCT	2020	20/20	51.5 ± 17.0, 55.1 ± 18.8	14/6, 11/9	1.9 ± 2.1m, 1.6 ± 1.5 m	10/10, 11/9	rTMS+CT, sham+CT	Figure-eight coil (Yiruide Medical Equipment Company, China)	Ipsilesional M1	10 Hz, 90% RMT, 1500 pulses	2 weeks, 7 days per week	VAS, FMA-UE
Sun et al. (2019)	RCT	2019	20/20	48.1 ± 8.5, 50.1 ± 7.7	15/5, 12/8	6.0 ± 1.5 d, 7.0 ± 1.1 d	8/12, 10/10	rTMS+CT, sham+CT	Figure-eight coil (CCY-1 stimulator, Yiruide Medical Equipment Company, China)	Ipsilesional M1	10 Hz, 80% RMT, 1500 pulses	4 weeks, 6 days per week	VAS, MEP
De Oliveira et al. (2014)	RCT	2014	12, 11	55.0 ± 9.67, 57.8 ± 11.86	5/7, 6/6	64.1 ± 49.2 m, 50.1 ± 28.0 m	4/8, 2/9	rTMS+CT, sham+CT	Figure-eight coil (MagPROX100 machine Magventure Tonika Elektronic, Farum, Denmark)	Left DLPFC/ PMC	10 Hz, 120% RMT, 1250 pulses	2 weeks, 5 days per week	VAS, MPQ, HAM-D, HAM-A

HADM, Hamilton Rating Scale for Depression; HAMA, Hamilton Anxiety Scale; T, Treatment Group; C, Control Group; rTMS, Repetitive Transcranial Magnetic Stimulation; RCT, Randomized Controlled Trial; VAS, M1, motor cortical area; MEP, Motor-Evoked Potential; RMT, Resting Motor Threshold; NRS, Numeric rating scale; SF-MPQ-2, Short-form McGill Pain Questionnaire-2; MPQ-2, McGill Pain Questionnaire; VAS, visual analog scale; CT, conventional therapy; d, day; m, month; FMA-UE, Fugl-Meyer Assessment Upper Extremity Scale; /, no information.

### 3.3 Risk of bias

**Figure 2:**
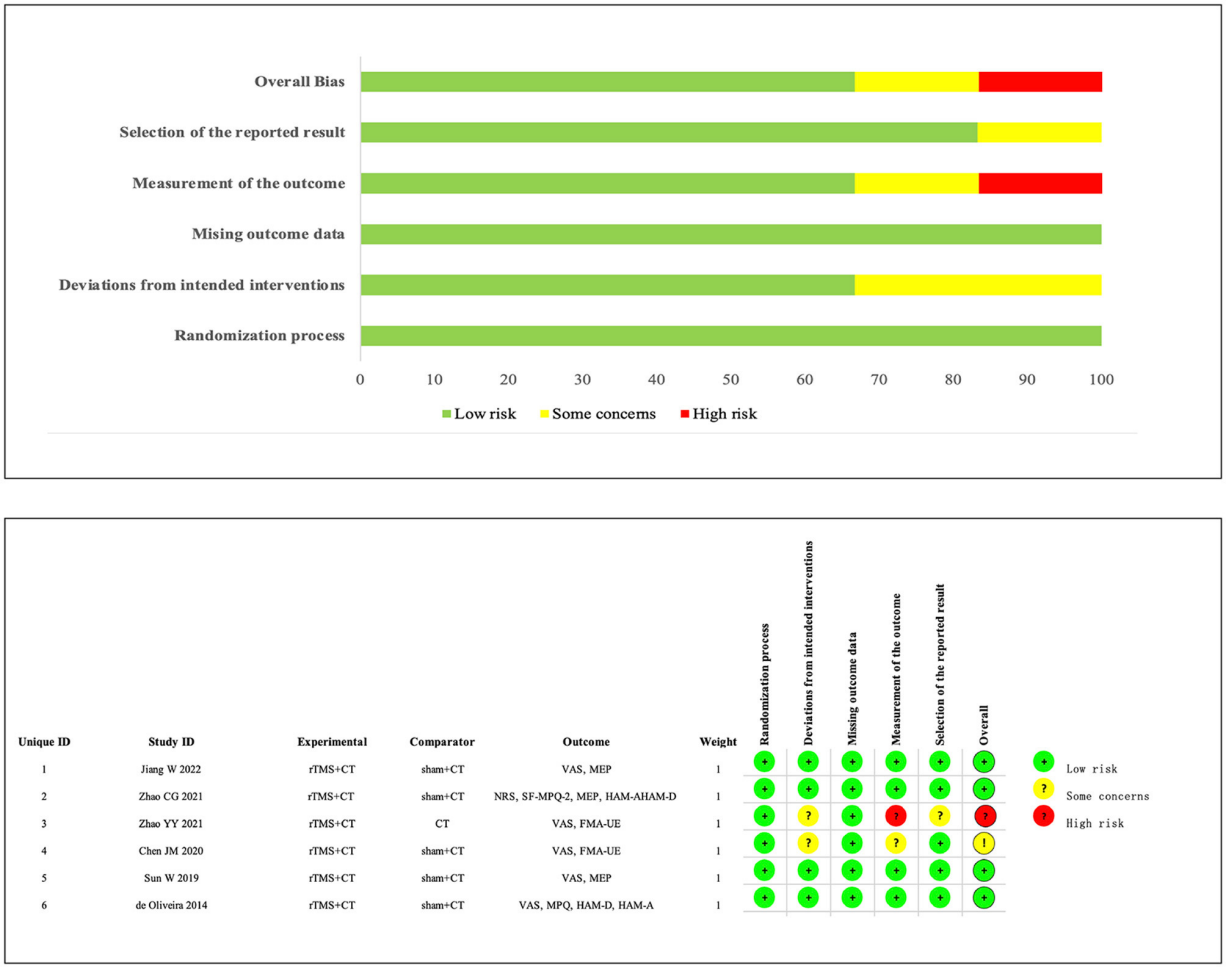
Risk of Bias of RCTs.

We assessed the risk of bias in the six RCTs using Cochrane RoB 2.0. One study was rated as high risk, one study had some concerns, and the remaining four studies were assessed as low risk. Simultaneously, using the Pedro scale, four studies scored ≥7 points, indicating high-quality research. Two studies scored 6 points, categorizing them as medium-quality studies (Moseley et al., 2020; Meng et al., 2023). The specific bias risks are detailed in Figure 2, Table 2.

**Table 2:** PEDro scores of the included studies.

**References**	**Eligibility criteria**	**Random allocation**	**Concealedallocation**	**Baseline comparability**	**Participantsblinded**	**Therapists blinded**	**Assessors blinded**	**Adequate follow-up**	**No missing data or intention to treat analysis**	**Between-groups comparisons**	**Point estimates and variability**	**Total score (/10)**
Jiang et al. (2022)	Yes	1	0	1	1	1	1	1	1	1	1	9
Zhao C.-G. et al. (2021)	Yes	1	1	1	1	0	1	1	1	1	1	9
Zhao Y. Y. et al. (2021)	Yes	1	0	1	0	0	0	1	1	1	1	6
Chen et al. (2020)	Yes	1	0	1	0	0	0	1	1	1	1	6
Sun et al. (2019)	Yes	1	0	1	0	0	0	1	1	1	1	9
De Oliveira et al. (2014)	Yes	1	0	1	1	0	1	1	1	1	1	8

1 = Yes, 0 = No.

### 3.4 Results of the meta-analysis

**Figure 3:**
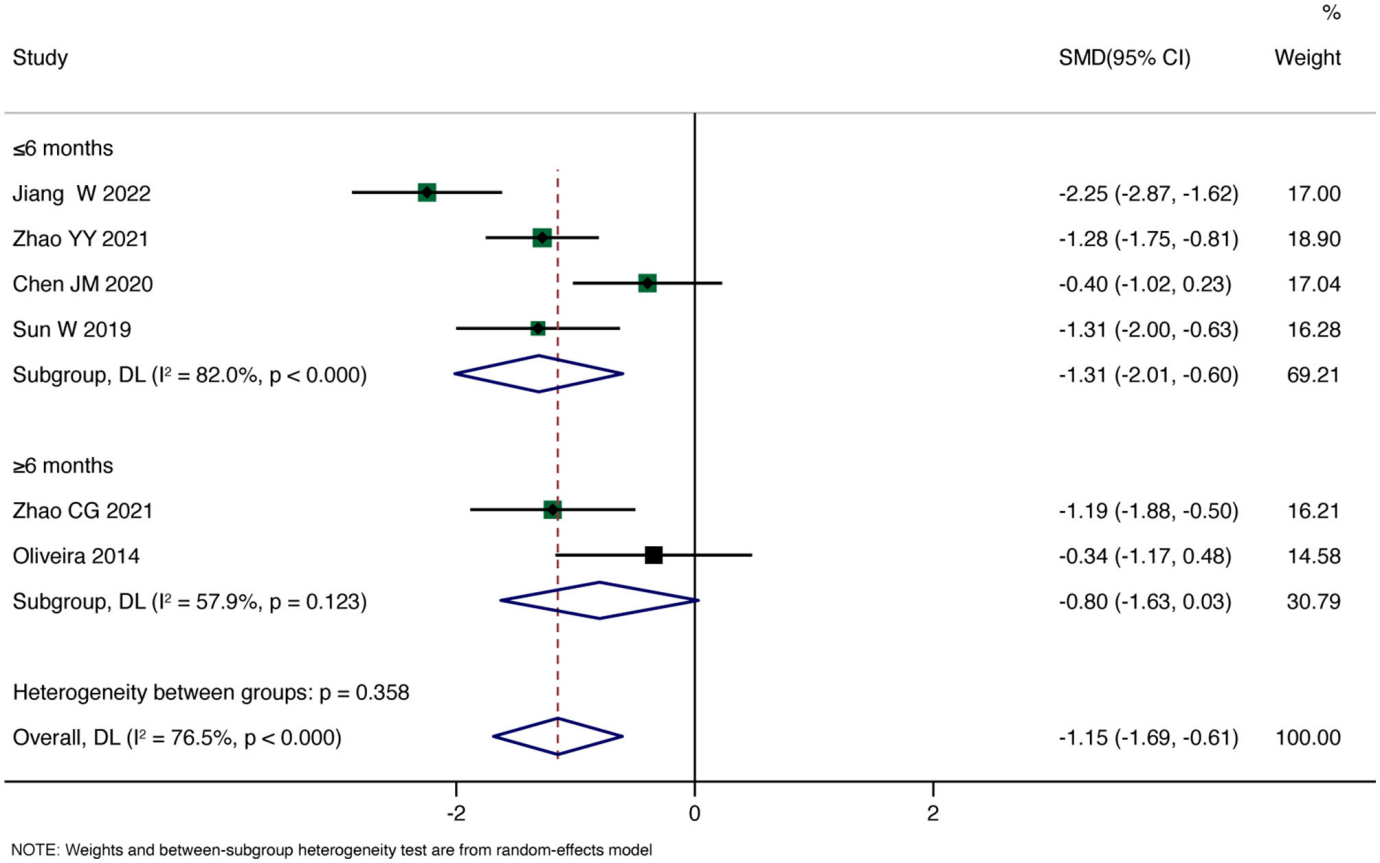
Forest plot for pain.

Primary Outcome: Six studies reported pain scores. The overall pooled Standardized Mean Difference (SMD) using a random-effects model showed that rTMS significantly reduced patients' pain scores compared to the control group (SMD = −1.15, 95% CI: −1.69, −0.61, *P* < 0.001). However, there was substantial heterogeneity (I^2^ = 71.5%, *P* < 0.001). Subgroup analysis for the duration less than 6 months revealed a significant reduction in pain scores with rTMS (SMD = −1.31, 95% CI: −2.01, −0.60, *P* < 0.001). In contrast, for the subgroup with a duration >6 months, the analysis showed no significant effect of rTMS on improving patients' pain (SMD = −0.80, 95% CI: −1.63, 0.03, *P* = 0.059). Detailed results are shown in Figure 3.

**Figure 4:**
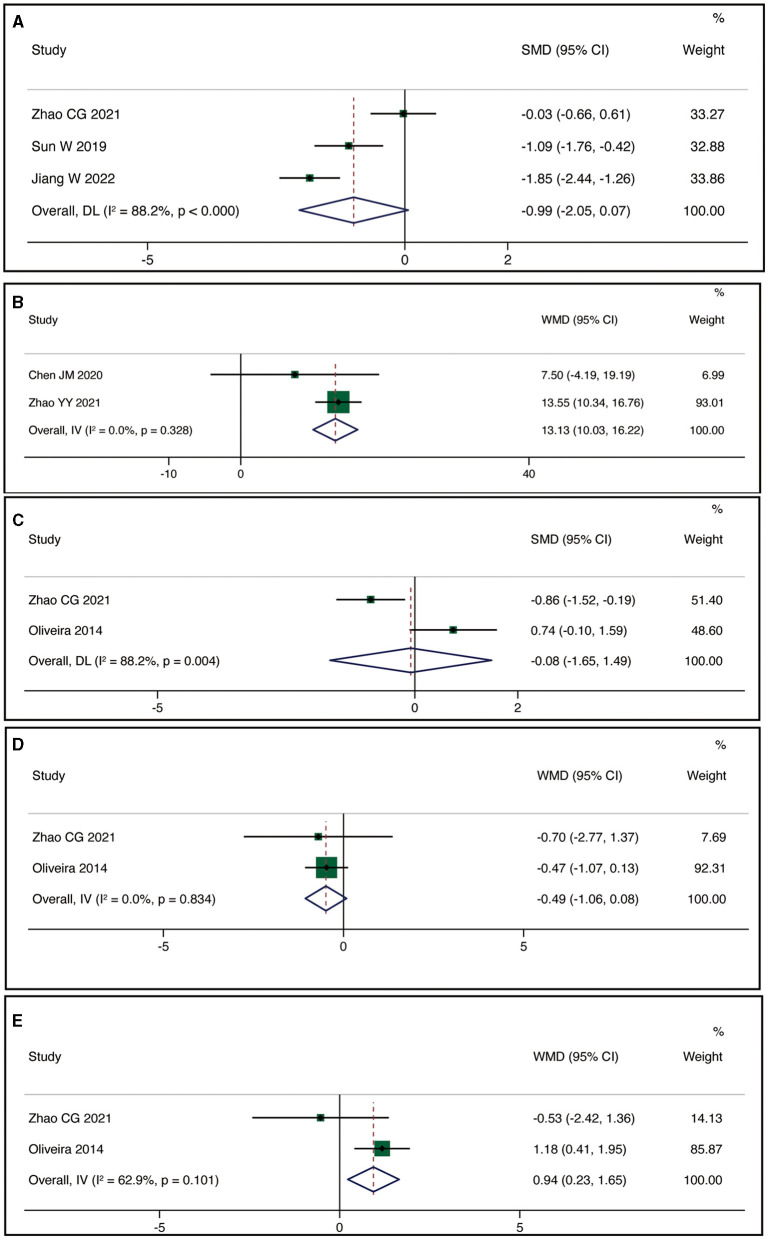
Forest plot for MEP-latency **(A)**, FMA-UE **(B)**, MPQ **(C)**, HAM-A **(D)**, HAM-D **(E)**.

Three studies reported MEP-latency. The meta-analysis indicated no statistically significant difference between the rTMS group and the control group (SMD = −0.99, 95% CI:−2.05, 0.07, *P* = 0.066), but with high heterogeneity (I^2^ = 88.2%, *P* < 0.001) (Figure 4A). Two studies reported FMA-UE. The meta-analysis demonstrated a statistically significant difference in improving FMA-UE scores between the rTMS group and the control group (WMD = 13.13, 95% CI: 10.03, 16.22, *P* < 0.001) with low heterogeneity (I^2^ = 0%, *P* = 0.328) (Figure 4B). Two studies reported MPQ. Using a random-effects model, the meta-analysis revealed no statistically significant difference in improving patients' MPQ scores between the rTMS group and the control group (SMD = −0.08, 95% CI: −1.65, 1.49, *P* = 0.921), but with high heterogeneity (I^2^ = 88.2%, *P* = 0.004) (Figure 4C). Two studies reported HAM-A. The meta-analysis showed no statistically significant difference in reducing patients' HAM-A scores between the rTMS group and the control group (WMD = −0.49, 95% CI: −1.06, 0.08, *P* = 0.095) with low heterogeneity (I^2^ = 0%, *P* = 0.834) (Figure 4D). Two studies reported HAM-D. The meta-analysis indicated no statistically significant difference in improving patients' HAM-D scores between the rTMS group and the control group (WMD = 0.95, 95% CI: 0.23, 1.65, *P* = 0.010) with high heterogeneity (I^2^ = 62.9%, *P* = 0.101) (Figure 4E).

### 3.5 Sensitivity analysis and publication bias

**Figure 5:**
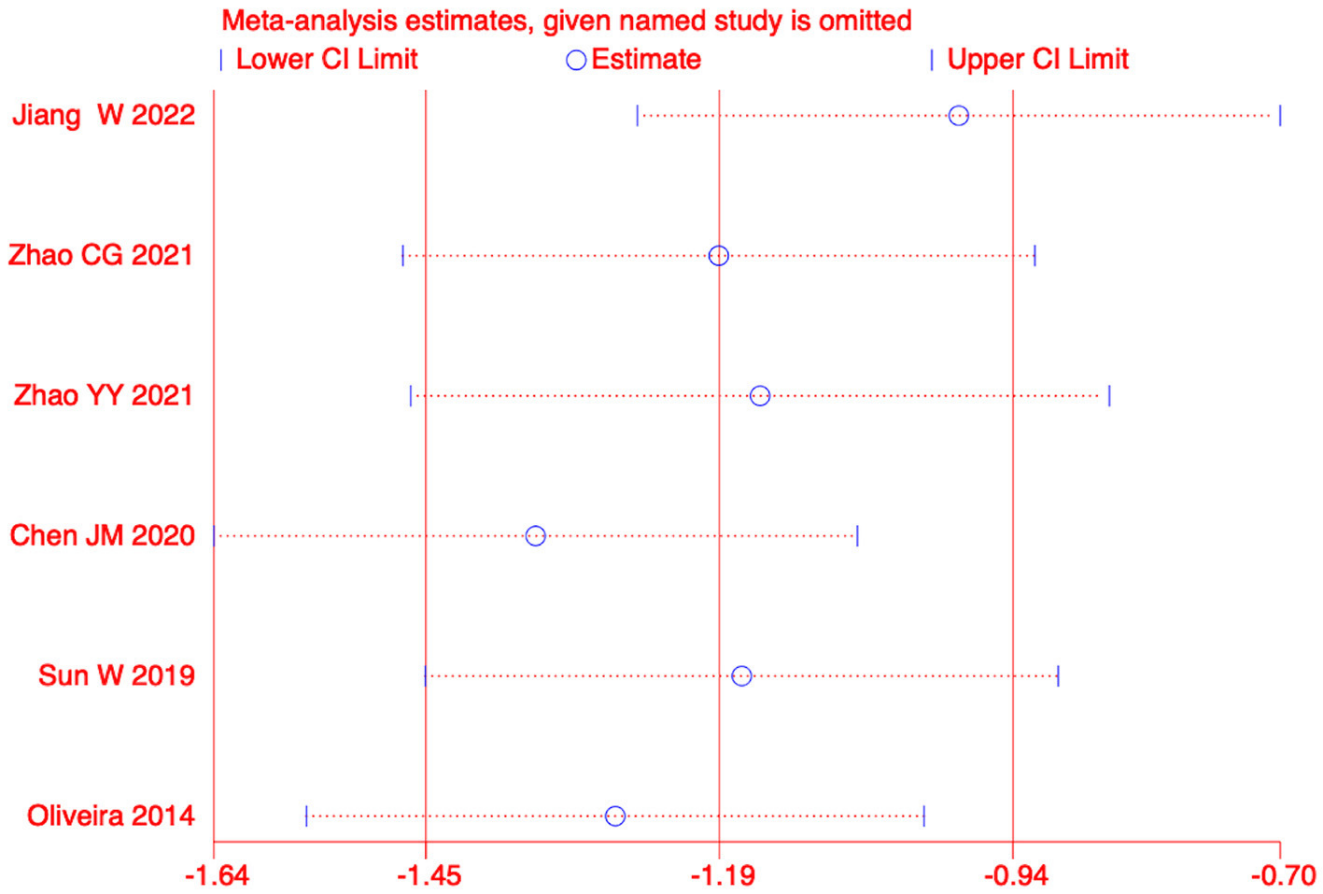
Sensitivity analysis of pain.

**Figure 6:**
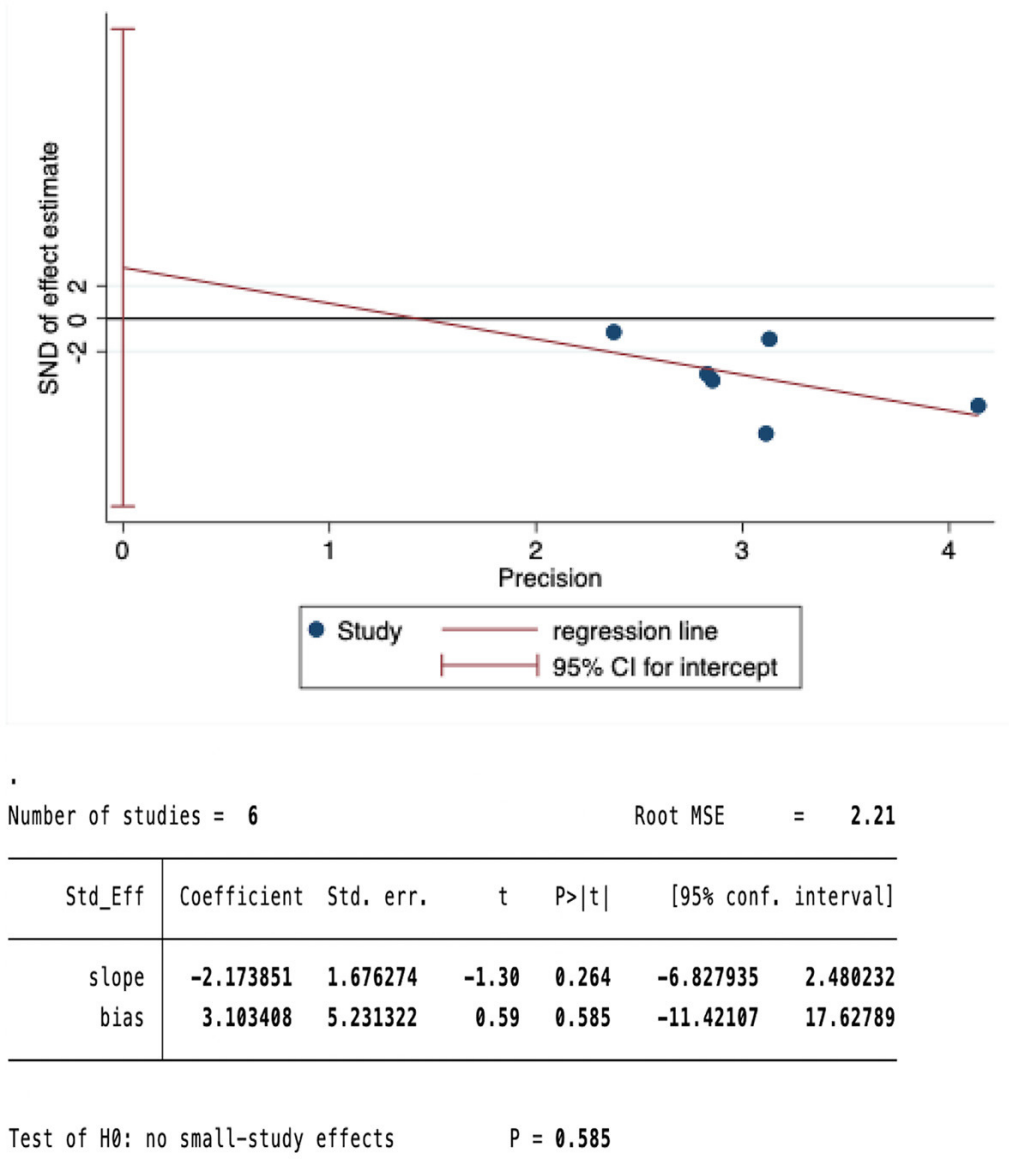
Egger test of pain.

We conducted a sensitivity analysis on the meta-analysis results of the primary outcomes (effect = −1.19, CI: −1.45, −0.93), Indicating that the results are stable. The results of Egger test were P=0.585>0.05, suggesting no significant publication bias (Figures 5, 6).

## 4 Discussion

Our meta-analysis results integrated quantitative data from pain, anxiety, and depression rating scales, as well as FMA-UE and MEP-latency assessments in CPSP patients treated with rTMS. The data analysis revealed a significant efficacy of rTMS in alleviating pain and enhancing upper limb motor function in CPSP patients. Nevertheless, there was no statistically significant improvement noted in the patients' anxiety and depression levels, and MEP-latency remained unaffected.

The pain inhibitory mechanisms in CPSP patients may be dysregulated. The presence of post-stroke lesions results in decreased excitability of the affected hemisphere's M1, leading to reduced neural output, including interhemispheric inhibition (IHI) from the unaffected hemisphere to M1. This results in a relative increase in excitability of the contralateral hemisphere's M1 and increased neural output, thereby shifting IHI from the contralateral hemisphere's M1 to the affected hemisphere's M1, inhibiting the excitability of the affected hemisphere's M1 (Gerges et al., 2022). A recent study found that rTMS induces an increase in IHI from the affected hemisphere to the contralateral hemisphere, thereby alleviating pain (Alhassani et al., 2019). Thus, LF-rTMS over the unaffected hemisphere may reduce inhibition of the affected hemisphere. Conversely, HF-rTMS over the affected hemisphere increases inhibition of the unaffected hemisphere, normalizing cortical excitability and ultimately achieving pain relief.

Numerous clinical trials have established that a reduction in gamma-aminobutyric acid GABAergic neurotransmission within the central nervous system is a principal etiology of persistent neuropathic pain (Yang et al., 2019; Lanza et al., 2020). It is widely posited that corticosterogenic inhibition within the M1, known as intracortical inhibition (ICI), mirrors the activity of interneurons. Both ICI and intracortical facilitation (ICF) are considered potential indicators of GABAergic inhibitory interneuron function, particularly in relation to GABAergic processes. Prior investigations have demonstrated that high-frequency rTMS can enhance ICI and ICF, with these alterations being correlated with pain alleviation in patients with CPSP (Hosomi et al., 2013). Consequently, rTMS may exert its analgesic effect on CPSP through a mechanism that involves the augmentation of GABAergic neuronal transmission (Pan et al., 2022).

rTMS may be mechanistically analogous to Motor Cortex Stimulation (MCS), as indicated by findings from MCS research. These investigations propose that MCS could directly modulate regions of the brain involved in the affective processing of pain, and/or indirectly initiate mechanisms enhancing the activity of inhibitory pathways in the dorsal horn (Leung et al., 2009), Additionally, rTMS might mitigate pain by augmenting perfusion to the afflicted area. Evidence indicates a relative decrease in Cerebral Blood Flow (CBF) in chronic pain conditions, with PET studies revealing that rTMS application targeting the M1 significantly elevates CBF in individuals with neuropathic pain (Jin et al., 2015; Quesada et al., 2018). Recent studies have reported the potential of rTMS in alleviating neuropathic pain in conditions such as post-spinal cord injury, post-trigeminal nerve surgery pain, and burning mouth syndrome (Ma et al., 2015; Gatzinsky et al., 2021; Isagulyan et al., 2023). The M1 has been suggested as an effective target for pain relief (O'Brien et al., 2016), and functional neuroimaging studies indicate that rTMS applied to the pre-motor cortex (PMC)/Dorsolateral Prefrontal Cortex (DLPFC) can provide robust and lasting analgesic effects, improving the condition of patients with severe depression (Ciampi Andrade et al., 2014; Che et al., 2021; Zhu et al., 2022). While previous research predominantly associated CPSP with thalamic damage, recent studies propose that vascular damage in any part of the central nervous system can lead to CPSP (Flaster et al., 2013; Cheng et al., 2023). This shift in understanding may be attributed to post-stroke abnormalities in pathways such as the corticospinal tract, thalamocortical tract, spinothalamic tract, and posterior limb of the internal capsule, ultimately resulting in abnormal neural excitations associated with pain (De Oliveira et al., 2012; Osama et al., 2018).

In clinical settings, the pharmacological management of CPSP typically involves the trial of various medications until pain relief is achieved, often requiring combinations of multiple drugs. Initial therapy for neuropathic pain typically involves tricyclic antidepressants, such as amitriptyline (75 mg/day), which effectively reduces pain in CPSP patients (Kremer et al., 2016; Obata, 2017). Adverse effects, including fatigue and dry mouth, are commonly reported, particularly with plasma concentrations exceeding 300 nmol/L (Dworkin et al., 2007). Anticonvulsant drugs, such as gabapentin and pregabalin, are known for their efficacy in both peripheral and central neuropathic pain by reducing neuronal hyperexcitability. Pregabalin has shown significant therapeutic benefits in pain intensity for central neuropathic pain patients, with common adverse effects including nausea, somnolence, cognitive decline, and dizziness (Vranken et al., 2008). Lamotrigine has been found to be well-tolerated and beneficial for pain relief in CPSP patients (Dworkin et al., 2007; Rollo et al., 2023).

Studies have indicated that in the treatment of CPSP, transcranial magnetic TMS with a frequency >5 Hz is more effective than low-frequency stimulation. This effectiveness may be attributed to the ability of high-frequency stimulation to restore the excitability of the abnormal cortex (Khedr et al., 2005; Kobayashi et al., 2015). Alhassani et al. (2019) discovered that the affected M1 on the contralateral side inhibits the unaffected M1. Therefore, high-frequency rTMS to the damaged hemisphere increases inhibition of the unaffected hemisphere, normalizing cortical excitability, and producing pain relief. Although the mechanisms underlying the analgesic effects of rTMS on M1 are not fully understood, they likely involve several factors. Firstly, rTMS can alter cortical excitability, and existing evidence suggests that the pain relief associated with rTMS in post-stroke pain is often accompanied by the restoration of cortical excitability abnormalities (Hosomi et al., 2013). Secondly, rTMS induces neuroplastic changes in the brain by mediating the up-down regulatory mechanism of cortical-spinal inhibition, ultimately leading to increased secretion of brain-derived neurotrophic factor (BDNF) (Zhao C.-G. et al., 2021). This process also influences the structural and functional connections of brain regions involved in pain processing and modulation (Dall'Agnol et al., 2014; Pan et al., 2022). Subgroup analysis in this study revealed that patients with CPSP lasting more than 6 months showed no significant relief in pain compared to the control group. This suggests that the mechanisms of pain in patients with a longer duration of illness may be more complex and require further research for clarification.

## 5 Limitations

Several limitations should be acknowledged in our study. Firstly, this meta-analysis is based on a limited pool of six randomized controlled trials (RCTs), each with a sample size < 100. This small sample size may potentially exaggerate the treatment effects. Secondly, only two studies reported measurements of depression and anxiety. Caution should be exercised in interpreting these results due to the limited data available. The efficacy differences between different stimulation sites (M1 vs. DLPFC) were not analyzed in our study due to the insufficient number of included studies. Future research may benefit from comparing the effects of different intervention sites (M1 vs. DLPFC) on CPSP patients. Moreover, multicenter, randomized controlled, double-blind trials with diverse stimulation protocols are warranted in clinical practice. These trials would facilitate longitudinal and cross-sectional comparisons between different stimulation parameters to determine the optimal stimulation protocol.

## 6 Conclusion

Our systematic review and meta-analysis of rTMS for the treatment of CPSP indicate that rTMS may be effective in alleviating pain and potentially improving motor function in CPSP patients. However, its efficacy for depression, anxiety, and MEP-latency remains inconclusive.

## Data availability statement

The original contributions presented in the study are included in the article/Supplementary material, further inquiries can be directed to the corresponding author.

## Author contributions

YL: Data curation, Writing – original draft, Writing – review & editing. RM: Methodology, Supervision, Writing – review & editing. HZ: Software, Writing – review & editing. QH: Conceptualization, Writing – original draft, Writing – review & editing. SY: Resources, Software, Validation, Writing – original draft, Writing – review & editing. FZ: Conceptualization, Data curation, Formal analysis, Funding acquisition, Investigation, Methodology, Project administration, Resources, Software, Supervision, Validation, Visualization, Writing – original draft, Writing – review & editing.
