# Supplementary material for: Repetitive transcranial magnetic stimulation in central post-stroke pain: a meta-analysis and systematic review of randomized controlled trials
Source: Front Neurosci. 2024 Jun 12;18:1367649. doi: 10.3389/fnins.2024.1367649 (PMC11199869; doi:10.3389/fnins.2024.1367649)
Supplement: Supplementary file 1 [file Data_Sheet_1.docx]

| **Exclusion list** |  |  |
| --- | --- | --- |
| Recruitment challenges in stroke neurorecovery clinical trials | Ferreira,Isadora Santos et al. | No-RCTs |
| Efficacy and Safety of High-Frequency Repetitive Transcranial Magnetic Stimulation for Poststroke Depression: A Systematic Review and Meta-analysis | Liu, Chaomeng et al. | No-RCTs |
| Insular and anterior cingulate cortex deep stimulation for central neuropathic pain: Disassembling the percept of pain | Galhardoni, Ricardo et al. | Non-stroke patients |
| Transcranial direct current stimulation in inflammatory bowel disease patients modifies resting-state functional connectivity: A RCT | Neeb, Lars et al. | Non-stroke patients |
| Changes in motor cortical excitability in schizophrenia following transcranial direct current stimulation | Gordon, Pedro Caldana et al. | Non-stroke patients |
| Effects of high-frequency repetitive transcranial magnetic stimulation on reducing hemiplegic shoulder pain in patients with chronic stoke: a randomized controlled trial | Choi, Gyu-Sik, | No-CPSP |
| Challenges in Recruitment for the Study of Noninvasive Brain Stimulation in Stroke: Lessons from Deep Brain Stimulation | Potter-Baker, Kelsey A et al. | No-RCTs |
| High Frequency Repetitive Transcranial Magnetic Stimulation Therapy For Chronic Neuropathic Pain: A Meta-analysis | Jin, Yu et al. | No-CPSP |
| Management of Central Poststroke Pain: Systematic Review of Randomized Controlled Trials | Mulla, Sohail M et al. | No-RCTs |
| Repetitive transcranial magnetic stimulation in cervical dystonia: effect of site and repetition in a randomized pilot trial | Pirio Richardson, Sarah et al. | Non-stroke patients |
| Results availability for analgesic device, complex regional pain syndrome, and post-stroke pain trials: comparing the RReADS, RReACT, and RReMiT databases | Dufka, Faustine L et al. | No-RCTs |
| Cutaneous anesthesia of the forearm enhances sensorimotor function of the hand | Petoe, Matthew A et al. | Non-stroke patients |
| Stimulation of primary motor cortex and reorganization of cortical function | Saitoh, Youichi et al. | No-CPSP |
| Theta burst stimulation in the rehabilitation of the upper limb: a semirandomized, placebo-controlled trial in chronic stroke patients | Talelli, Penelope et al. | No-CPSP |
| Human motor plasticity induced by mirror visual feedback | Nojima, Ippei et al. | No-RCTs |
| The clinical aspects of mirror therapy in rehabilitation: a systematic review of the literature | Rothgangel, Andreas Stefan et al. | No-RCTs |
| Diffusion tensor fiber tracking in patients with central post-stroke pain; correlation with efficacy of repetitive transcranial magnetic stimulation | Ciampi de Andrade, Daniel et al. | No-RCTs |
| Safety of 6-Hz primed low-frequency rTMS in stroke | Carey, James R et al. | No-CPSP |
| Motor cortex stimulation for neuropathic pain | Lazorthes, Y et al. | No-CPSP |
| Technology insight: noninvasive brain stimulation in neurology-perspectives on the therapeutic potential of rTMS and tDCS | Fregni, Felipe et al. | No-CPSP |
| Motor cortex stimulation for intractable pain | Osenbach, Richard K. | Non-stroke patients |
| Recovery of motor function after stroke | Brown, Jeffrey A. | No-RCTs |
| Neurogenic pain relief by repetitive transcranial magnetic cortical stimulation depends on the origin and the site of pain | Lefaucheur, J-P et al. | No-CPSP |
| Interventional neurophysiology for pain control: duration of pain relief following repetitive transcranial magnetic stimulation of the motor cortex | Lefaucheur, J P et al. | No-RCTs |
| A Randomized, Sham-Controlled Trial of Repetitive Transcranial Magnetic Stimulation Targeting M1 and S2 in Central Poststroke Pain: A Pilot Trial | Ojala, Juhani et al. | Interventions were not satisfied |
| Efficacy of Repetitive Transcranial Magnetic Stimulation for Acute Central Post-stroke Pain: A Case Study | Malfitano, Calogero et al. | No-RCTs |
| Clinical effect of repeated transcranial magnetic stimulation on 20 cases of CPSP | Chen Huizhen et al. | Data missing |
| Effect of repeated transcranial magnetic stimulation combined with carbamazepine in the treatment of central pain after stroke | Li Na et al. | Data missing |
| Effect of repeated transcranial magnetic stimulation on symptom improvement of central pain after stroke | Liu Ting et al. | Data missing |
| Clinical efficacy of repeated transcranial magnetic stimulation in the treatment of central pain after stroke | Shen Yiwen et al. | Interventions were not satisfied |

|  |
| --- |

| \| The search strategy \| \| --- \| \| Pubmed search strategy \| \| #1 Stroke[MeSH Terms]) OR Cerebrovascular Accident OR Cerebrovascular Accidents OR CVA (Cerebrovascular Accident) OR CVAs (Cerebrovascular Accident) OR Cerebrovascular Apoplexy OR Apoplexy, Cerebrovascular OR Vascular Accident, Brain OR Brain Vascular Accident OR Brain Vascular Accidents OR Vascular Accidents, Brain OR Cerebrovascular Stroke OR Cerebrovascular Strokes OR Stroke, Cerebrovascular OR Strokes, Cerebrovascular OR Apoplexy OR Cerebral Stroke OR Cerebral Strokes OR Stroke, Cerebral OR Strokes, Cerebral OR Stroke, Acute OR Acute Stroke OR Acute Strokes OR Strokes, Acute OR Cerebrovascular Accident, Acute OR Acute Cerebrovascular Accident OR Acute Cerebrovascular Accidents OR Cerebrovascular Accidents, Acute) \| \| #2 (Repetitive Transcranial Magnetic Stimulation[MeSH Terms]) OR (Transcranial Magnetic Stimulation) OR (TMS) OR (rTMS) OR (iTBS) OR (theta burst transcranial magnetic stimulation) \| \| #3 ("Pain"[Mesh]) OR (central neuropathic pain)) OR (central pain)) OR (neuropathic pain)) OR (Ache) \| \| #4 #1 AND #2 AND #3 \| \| EMBASE search strategy \| \| #1 'stroke'/exp OR stroke OR 'cerebrovascular accident'/exp OR 'cerebrovascular accident' OR (cerebrovascular AND ('accident'/exp OR accident)) \| \| #2 'repetitive transcranial magnetic stimulation'/exp OR 'repetitive transcranial magnetic stimulation' OR (repetitive AND transcranial AND magnetic AND ('stimulation'/exp OR stimulation)) OR 'transcranial magnetic stimulation'/exp OR 'transcranial magnetic stimulation' OR (transcranial AND magnetic AND ('stimulation'/exp OR stimulation)) \| \| #3 'pain'/exp OR pain OR ache \| \| #4 #1 AND #2 AND #3 \| \| WOS search strategy \| \| #1 (TS=(stroke) OR TS=(Cerebrovascular Accident )) OR TS=(Cerebrovascular Accident ) \| \| #2 ((TS=(Repetitive Transcranial Magnetic Stimulation)) OR TS=( Transcranial Magnetic Stimulation)) OR TS=(TMS) \| \| #3 (((TS=(Pain)) OR TS=(central neuropathic pain)) OR TS=(central pain)) OR TS=(AChe) \| \| #4 #1 AND #2 AND #3 \| \| Cochrane Library search strategy \| \| #1 (stroke):ti,ab,kw" OR ("cerebrovascular accident"):ti,ab,kw \| \| #2 (Repetitive Transcranial Magnetic Stimulation):ti,ab,kw" OR ( Transcranial Magnetic Stimulation):ti,ab,kw" \| \| #3 (Pain):ti,ab,kw" OR (central neuropathic pain):ti,ab,kw" OR (central pain):ti,ab,kw" \| \| #4 #1 AND #2 AND #3 \| |
| --- | --- | --- | --- | --- | --- | --- | --- | --- | --- | --- | --- | --- | --- | --- | --- | --- | --- | --- | --- | --- | --- |

**PRISMA 2020**

| **Section and Topic** | **Item #** | **Checklist item** | **Location where item is reported** |
| --- | --- | --- | --- |
| **TITLE** | | |  |
| Title | 1 | Identify the report as a systematic review. | 1 |
| **ABSTRACT** | | |  |
| Abstract | 2 | See the PRISMA 2020 for Abstracts checklist. | 1 |
| **INTRODUCTION** | | |  |
| Rationale | 3 | Describe the rationale for the review in the context of existing knowledge. | 2 |
| Objectives | 4 | Provide an explicit statement of the objective(s) or question(s) the review addresses. | 2 |
| **METHODS** | | |  |
| Eligibility criteria | 5 | Specify the inclusion and exclusion criteria for the review and how studies were grouped for the syntheses. | 3 |
| Information sources | 6 | Specify all databases, registers, websites, organisations, reference lists and other sources searched or consulted to identify studies. Specify the date when each source was last searched or consulted. | 3 |
| Search strategy | 7 | Present the full search strategies for all databases, registers and websites, including any filters and limits used. | 4 |
| Selection process | 8 | Specify the methods used to decide whether a study met the inclusion criteria of the review, including how many reviewers screened each record and each report retrieved, whether they worked independently, and if applicable, details of automation tools used in the process. | 4 |
| Data collection process | 9 | Specify the methods used to collect data from reports, including how many reviewers collected data from each report, whether they worked independently, any processes for obtaining or confirming data from study investigators, and if applicable, details of automation tools used in the process. | 4 |
| Data items | 10a | List and define all outcomes for which data were sought. Specify whether all results that were compatible with each outcome domain in each study were sought (e.g. for all measures, time points, analyses), and if not, the methods used to decide which results to collect. | 4 |
|  | 10b | List and define all other variables for which data were sought (e.g. participant and intervention characteristics, funding sources). Describe any assumptions made about any missing or unclear information. | Table 1 |
| Study risk of bias assessment | 11 | Specify the methods used to assess risk of bias in the included studies, including details of the tool(s) used, how many reviewers assessed each study and whether they worked independently, and if applicable, details of automation tools used in the process. | 4 |
| Effect measures | 12 | Specify for each outcome the effect measure(s) (e.g. risk ratio, mean difference) used in the synthesis or presentation of results. | 5 |
| Synthesis methods | 13a | Describe the processes used to decide which studies were eligible for each synthesis (e.g. tabulating the study intervention characteristics and comparing against the planned groups for each synthesis (item #5)). | 5 |
|  | 13b | Describe any methods required to prepare the data for presentation or synthesis, such as handling of missing summary statistics, or data conversions. | 5 |
|  | 13c | Describe any methods used to tabulate or visually display results of individual studies and syntheses. | 5 |
|  | 13d | Describe any methods used to synthesize results and provide a rationale for the choice(s). If meta-analysis was performed, describe the model(s), method(s) to identify the presence and extent of statistical heterogeneity, and software package(s) used. | 5 |
|  | 13e | Describe any methods used to explore possible causes of heterogeneity among study results (e.g. subgroup analysis, meta-regression). | 5 |
|  | 13f | Describe any sensitivity analyses conducted to assess robustness of the synthesized results. | 5 |
| Reporting bias assessment | 14 | Describe any methods used to assess risk of bias due to missing results in a synthesis (arising from reporting biases). | 5 |
| Certainty assessment | 15 | Describe any methods used to assess certainty (or confidence) in the body of evidence for an outcome. | Table 2 |
| **RESULTS** | | |  |
| Study selection | 16a | Describe the results of the search and selection process, from the number of records identified in the search to the number of studies included in the review, ideally using a flow diagram. | 5 |
|  | 16b | Cite studies that might appear to meet the inclusion criteria, but which were excluded, and explain why they were excluded. | Figure 2 |
| Study characteristics | 17 | Cite each included study and present its characteristics. | Table 1 |
| Risk of bias in studies | 18 | Present assessments of risk of bias for each included study. | Figure 2 |
| Results of individual studies | 19 | For all outcomes, present, for each study: (a) summary statistics for each group (where appropriate) and (b) an effect estimate and its precision (e.g. confidence/credible interval), ideally using structured tables or plots. | Figure 3, 4 |
| Results of syntheses | 20a | For each synthesis, briefly summarise the characteristics and risk of bias among contributing studies. | 5 |
|  | 20b | Present results of all statistical syntheses conducted. If meta-analysis was done, present for each the summary estimate and its precision (e.g. confidence/credible interval) and measures of statistical heterogeneity. If comparing groups, describe the direction of the effect. | 6 |
|  | 20c | Present results of all investigations of possible causes of heterogeneity among study results. | 6 |
|  | 20d | Present results of all sensitivity analyses conducted to assess the robustness of the synthesized results. | Figure 5 |
| Reporting biases | 21 | Present assessments of risk of bias due to missing results (arising from reporting biases) for each synthesis assessed. | 6 |
| Certainty of evidence | 22 | Present assessments of certainty (or confidence) in the body of evidence for each outcome assessed. | Table 2 |
| **DISCUSSION** | | |  |
| Discussion | 23a | Provide a general interpretation of the results in the context of other evidence. | 7 |
|  | 23b | Discuss any limitations of the evidence included in the review. | 7 |
|  | 23c | Discuss any limitations of the review processes used. | 8 |
|  | 23d | Discuss implications of the results for practice, policy, and future research. | 8 |
| **OTHER INFORMATION** | | |  |
| Registration and protocol | 24a | Provide registration information for the review, including register name and registration number, or state that the review was not registered. | 2 |
|  | 24b | Indicate where the review protocol can be accessed, or state that a protocol was not prepared. | 2 |
|  | 24c | Describe and explain any amendments to information provided at registration or in the protocol. | None |
| Support | 25 | Describe sources of financial or non-financial support for the review, and the role of the funders or sponsors in the review. | 9 |
| Competing interests | 26 | Declare any competing interests of review authors. | 9 |
| Availability of data, code and other materials | 27 | Report which of the following are publicly available and where they can be found: template data collection forms; data extracted from included studies; data used for all analyses; analytic code; any other materials used in the review. | 9 |

*From:*  Page MJ, McKenzie JE, Bossuyt PM, Boutron I, Hoffmann TC, Mulrow CD, et al. The PRISMA 2020 statement: an updated guideline for reporting systematic reviews. BMJ 2021;372:n71. doi: 10.1136/bmj.n71

For more information, visit: <http://www.prisma-statement.org/>
